# International Clinical Trials in Latin American and Caribbean Countries: Research and Development to Meet Local Health Needs

**DOI:** 10.3389/fphar.2017.00961

**Published:** 2018-01-05

**Authors:** Ricardo E. da Silva, Angélica A. Amato, Dirce B. Guilhem, Marta R. de Carvalho, Maria R. C. G. Novaes

**Affiliations:** ^1^Office of Clinical Trials, Brazilian Health Regulatory Agency (Anvisa), Brasília, Brazil; ^2^Health Sciences, University of Brasília, Brasília, Brazil; ^3^School of Medicine, Health Sciences Education and Research Foundation, Brasília, Brazil

**Keywords:** clinical trials, drugs, noncommunicable diseases, neglected diseases, Latin America and the Caribbean

## Abstract

**Introduction:** Although international health research involves some benefits for the host countries, such as access to innovative treatments, the research itself may not be aligned with their communities' actual health needs.

**Objective:** To map the global landscape of clinical trials run in Latin American and Caribbean countries and discuss the addressing of local health needs in the agenda of international clinical trials.

**Methods:** The present study is a cross-sectional overview and used data referent to studies registered between 01/01/2014 and 12/31/2014 in the World Health Organization's (WHO) International Clinical Trials Registry Platform (ICTRP).

**Results:** Non-communicable diseases such as diabetes, cancer, and asthma—studies which were financed mainly by industries—were the conditions investigated most in the region of Latin America and the Caribbean. The neglected diseases, on the other hand, such as Chagas disease, and dengue, made up 1% of the total number of studies. Hospitals and nonprofit nongovernmental organizations prioritize resources for investigating new drugs for neglected diseases, such as Chagas disease and dengue.

**Conclusion:** The international multicenter clinical trials for investigating new drugs are aligned with the health needs of the region of Latin America and the Caribbean, when one considers the burden resulting from the non-communicable diseases in this region. However, the transmissible diseases, such as tuberculosis and AIDS, and the neglected diseases, such as Chagas disease and dengue, which have an important impact on public health in this region, continue to arouse little interest among the institutions which finance the clinical trials.

## Introduction

In the last two decades, the number of clinical trials conducted in Latin America has increased significantly (Motti, 2008), with a growth rate of 12% being observed between 2005 and 2012, one of the highest in the world (Drain et al., 2014). Besides expanding the investigators' knowledge, globalization of clinical trials also brings a variety of other benefits; examples include increases in the external validity of the research itself (that is, its ability to produce generalizable results) and investment in infrastructure in the host country. There are, however, risks, mainly when the study involves countries with social, economic, and health inequalities. There are also other issues to consider, such as whether the rights of those participating in the research are being fully protected (Annas, 2009; Da Silva et al., 2016).

Although global clinical trials involve some benefits, the fact remains that the trials may not be aligned with the health needs of local communities. These benefits can, furthermore, be unfair when they fail to outweigh the potential harm to the participants and communities involved. The medical care extended to the study participants during the trials may fall below the standard of that found in developed countries, and the drugs or therapies are not always made available to participants or their communities subsequent to their authorization for sale in North America or Europe. In short, sponsors may fail to treat research participants fairly during trials through the provision of adequate care, or fail to treat them fairly afterwards, through denying them a fair share of the resulting benefits or profits (Bhutta, 2002; Killen et al., 2002; Schulz-Baldes et al., 2007).

The so-called 10/90 gap in medical and health-related research came to the attention of researchers, policy-makers and bioethicists in 1996. This refers to the fact that a mere 10% of annual funding into health research targets the health needs of the poorest 90% of the world's population; while the remaining 90% of resources focusses on the health concerns of the richest 10% of the global population (Vidyasagar, 2006; Benatar and Singer, 2010; Miranda and Zaman, 2010).

Latin America and the Caribbean are among the regions affected the most by the so-called neglected diseases, such as Chagas disease, dengue, rabies, and schistosomiasis. These conditions are referred to as neglected because they affect populations which are poor: as a result, they arouse little interest among the institutions which finance research for investigating new drugs. These regions have few resources and poor infrastructure. There is difficulty in accessing the health services, potable water, food, or personal hygiene resources. There are problems related to the proliferation and control of disease vectors (Holveck et al., 2007; Dujardin et al., 2010).

This paper aims to map the global landscape of clinical trials run in Latin American and Caribbean countries and discuss the addressing of local health needs in the agenda of international clinical trials.

## Methods

### Design

The present study is a cross-sectional overview. Data is from International Clinical Trials Registry Platform (ICTRP) database. The period considered was the date of studies registered in the ICTRP between 01/01/2014 and 12/31/2014. The period of data collection was: 03/01/2014–06/31/2015.

### Selection criteria

We included clinical trials registered in the ICTRP that involved drug interventions in Latin American and Caribbean countries (Antigua and Barbuda, Argentina, Belize, Bolivia, Brazil, Chile, Colombia, Costa Rica, Dominica, Dominican Republic, Ecuador, El Salvador, Grenada, Guatemala, Guyana, Haiti, Honduras, Jamaica, Mexico, Nicaragua, Panama, Paraguay, Peru, Saint Kitts and Nevis, Saint Vincent and the Grenadines, Saint Lucia, Suriname, Trinidad and Tobago, Uruguay, and Venezuela).

Exclusion criteria were observational studies, devices and studies on medical procedures.

### Selected variables

Age group, study sponsor, development phase, data monitoring committee and health condition classified by the International Classification of Diseases (10th Revision) (ICD-10) (WHO, 2016).

The variable “data monitoring committee” was only obtained from The EU Clinical Trials Register, that is a primary registry in the WHO Registry Network (WHO, 2017g).

When searching for studies on the platform, it is necessary to identify the search terms and to select filters. In the site (http://www.who.int/ictrp/en/) the type of the chosen search was “advanced.” In the fields (title, condition, intervention, primary sponsor, and secondary ID) there was no use of search terms. All recruitment statuses and were selected. The search was performed for each country separately. The date of registration was 01/01/2014–12/31/2014.

No bias control procedure was used. There was no calculation of the sample size.

A pivot table (dynamic table in Microsoft Excel 2016) was created based on a dynamic data source to match the data of the variables.

### Analysis

The age of the study populations was classified using the National Institute of Health guidelines. Age filters include: 80 and over: 80+ years; Aged: 65+ years; Middle Aged: 45–64 years; Adult: 19–44 years; Adolescent: 13–18 years; Child: 6–12 years; Preschool Child: 2–5 years; Infant: 1–23 months; Newborn: birth-1 month (The National Institutes of Health, 2017).

The countries which make up the region of Latin America and the Caribbean are as defined by the World Bank (World Bank, 2017).

Study sponsor was classified according to the information on the organization's website. The ICTRP defines the primary sponsor as the “organization which takes responsibility for the initiation, management and/or financing of a clinical trial” (WHO, 2017a).

This study was approved by Research Ethics Committee of the Health Sciences College of the University of Brasília (Brazil).

## Results

**Figure 1:**
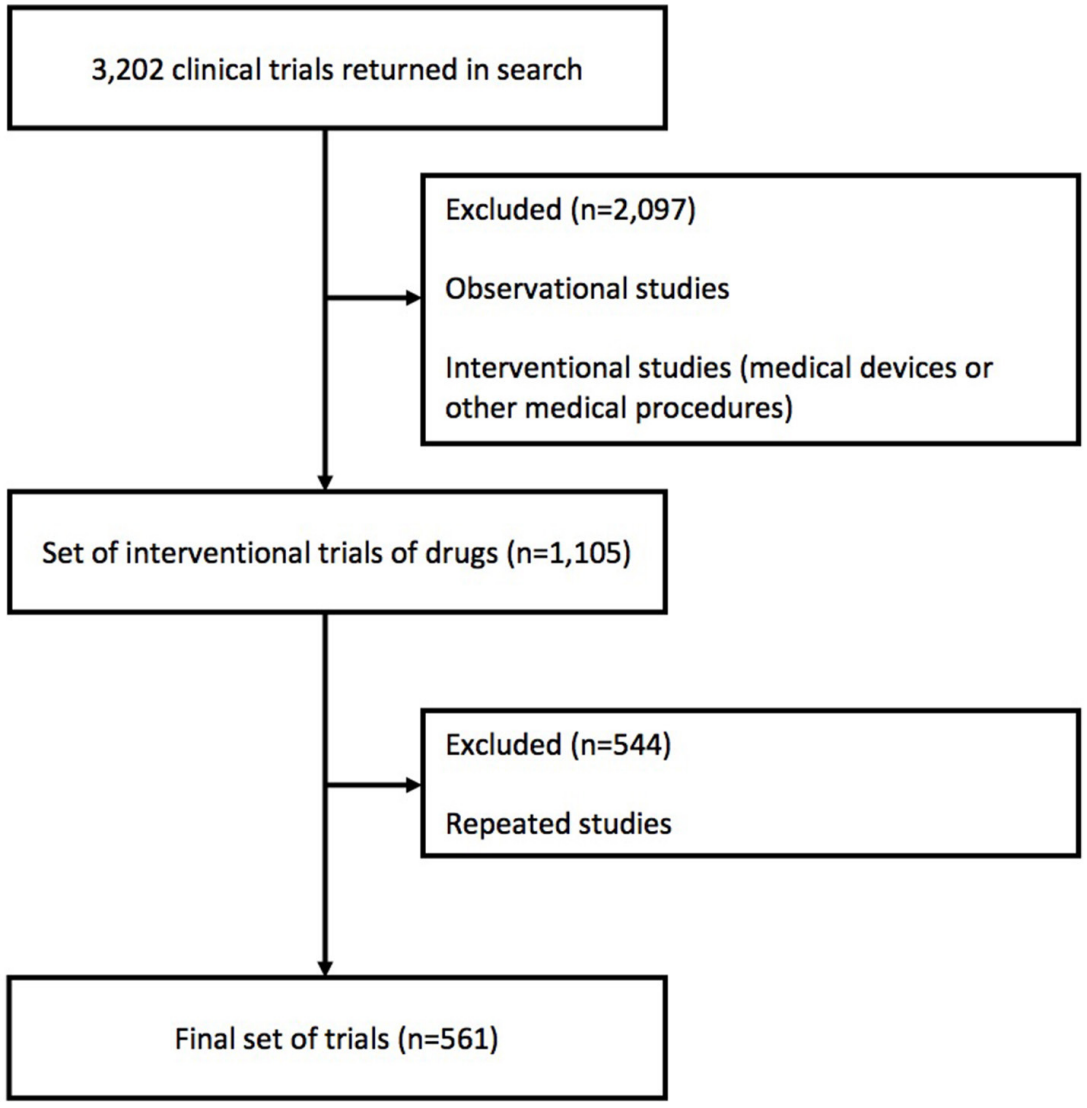
Study selection criteria. Adapted figure (Williams et al., 2015).

The search in the ICTRP returned 3,202 studies. After this, only drugs interventional studies were selected (*n* = 1,105). The number of registry entries meeting the inclusion criteria were: Argentina (191), Belize (1), Bolivia (2), Brazil (220), Chile (126), Colombia (97), Costa Rica (9), Dominican Republic (29), Ecuador (30), El Salvador (10), Guatemala (34), Haiti (1), Honduras (8), Mexico (229), Nicaragua (1), Panama (21), Paraguay (4), Peru (85), Uruguay (2), and Venezuela (4). Clinical trials which met the inclusion criteria were not found in Antigua and Barbuda, Dominica, Grenada, Guyana, Jamaica, Saint Kitts and Nevis, Saint Vincent and the Grenadines, Saint Lucia, Suriname or Trinidad, and Tobago. As the vast majority of these were multicenter clinical trials—that is, trials which took place in multiple countries at the same time—some studies inevitably were repeated. After eliminating these studies, the final set of trials was 561 (Figure 1).

Type 2 diabetes mellitus (E11), Seropositive rheumatoid arthritis (M05), Chronic obstructive pulmonary disease, unspecified (J44.9), Asthma (J45), and Malignant neoplasm of breast were studied most (Table 1).

**Table 1:** Diseases studied most in Latin America and the Caribbean (ICTRP, 2014).

**Diseases studied most, classified by International Classification of Diseases (ICD-10)**	**Number of studies**	**Percentage**
Type 2 diabetes mellitus (E11)	32	6
Seropositive rheumatoid arthritis (M05)	30	5
Chronic obstructive pulmonary disease, unspecified (J44.9)	27	5
Asthma (J45)	25	4
Malignant neoplasm of breast (C50)	22	4
Malignant neoplasm of bronchus and lung (C34)	14	2
Healthy volunteers	11	2
Unspecified human immunodeficiency virus [HIV] disease (B24)	08	1
Multiple sclerosis (G35)	08	1
Malignant neoplasm of prostate (C61)	07	1
Essential (primary) hypertension (I10)	07	1
Systemic lupus erythematosus (M32)	06	1
Plasmodium vivax malaria (B51)	05	1
Alzheimer disease (G30)	05	1
Acute ischaemic heart disease, unspecified (I24.9)	05	1
Heart failure (I50)	05	1
Other venous embolism and thrombosis (I82)	05	1
Ulcerative colitis (K51)	05	1
Juvenile arthritis (M08)	05	1

*Total number of studies in Latin America and the Caribbean: 561 (ICTRP, 2014)*.

The chronic conditions were studied most by the industry. On the other hand, hospitals and nonprofit nongovernmental organizations prioritized resources for investigating new drugs for neglected diseases, such as Chagas disease (Table 2). The neglected diseases—such as Chagas disease and dengue—corresponded to 1% of the total number of studies.

**Table 2:** Diseases studied most, by study sponsor in Latin America and the Caribbean (ICTRP, 2014).

**Study sponsor**	**Studied clinical condition**	**Number of studies**
Industry	Seropositive rheumatoid arthritis (M05)	27
	Chronic obstructive pulmonary disease, unspecified (J44.9)	26
	Asthma (J45)	25
	Type 2 diabetes mellitus (E11)	21
University	Type 2 diabetes mellitus (E11)	6
	Heart failure (I50)	3
	Other gastroenteritis and colitis of infectious and unspecified origin (A09)	1
	Viral and other specified intestinal infections (A08)	1
Hospital	Other acute post procedural pain (G89.18)	2
	Chagas disease (chronic) with heart involvement (B57.2)	1
	Sepsis due to other Gram-negative organisms (A41.5)	1
	Other gastroenteritis and colitis of infectious and unspecified origin (A09)	1
Nonprofit and Non-Governmental Organization	Plasmodium falciparum malaria (B50)	2
	Plasmodium vivax malária (B51)	2
	Chagas disease (B57)	1
	Respiratory tuberculosis, bacteriologically and histologically confirmed (A15)	1
Government	Necrotizing enterocolitis of fetus and newborn (P77)	2
	Plasmodium falciparum malaria (B50)	1
	Plasmodium vivax malaria (B51)	1
	Unspecified human immunodeficiency virus [HIV] disease (B24)	1

*Total number of studies in Latin America and the Caribbean: 561 (ICTRP, 2014)*.

Percentages of study sponsors: Industry (69%), University (12%), Hospital (5%), Government (2%), Nonprofit Organization (2%), others (10%).

**Figure 2:**
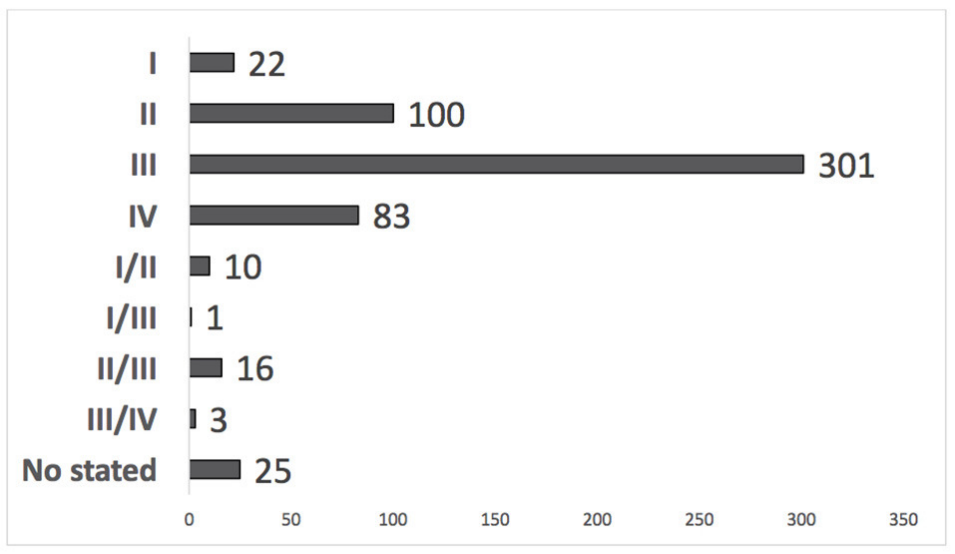
Number of clinical trials by development phase in Latin America and the Caribbean (ICTRP, 2014).

Among the studies, the most prevalent phase of trial was phase 3. Phase 1 trials were the least likely to be conducted (Figure 2).

**Figure 3:**
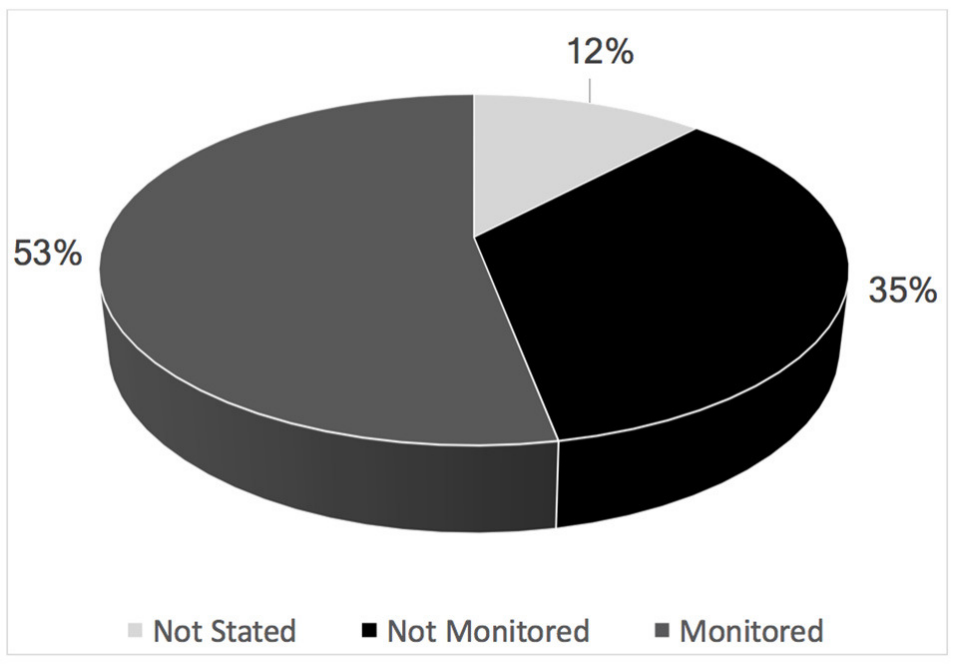
Percentage of clinical trials that were monitored or not by the data monitoring committee in Latin America and the Caribbean (ICTRP, 2014).

**Figure 4:**
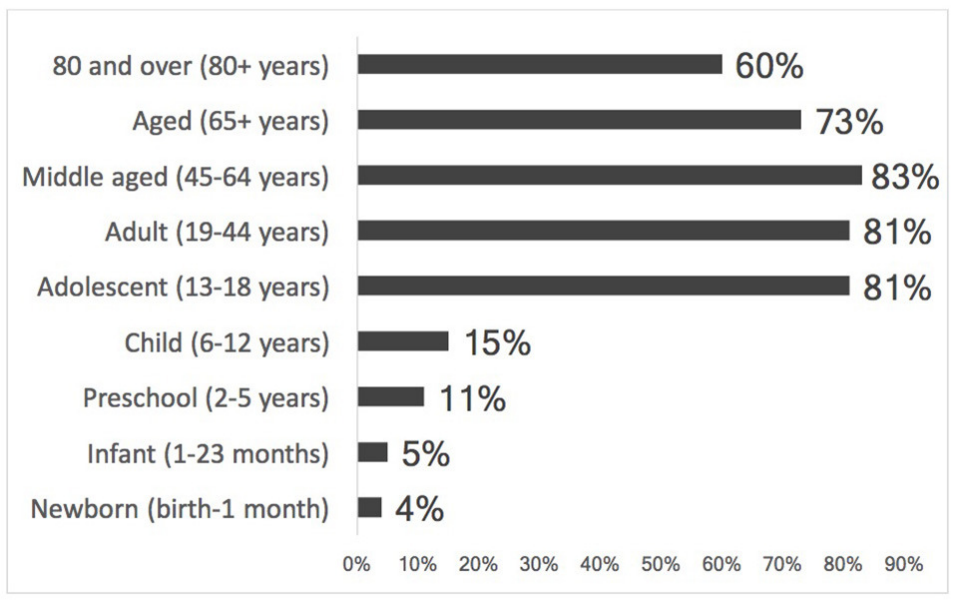
Percentage of clinical trials by age group in Latin America and the Caribbean (ICTRP, 2014).

In Latin America and the Caribbean, 53% of studies were monitored by a data monitoring committee (Figure 3). The age groups studied most were those that included the middle aged, adults, and adolescents. The pediatric group was studied least (Figure 4).

## Discussion

Diseases may be classified according to where they occur geographically, according to the investments in research and development, and according to their global burden. The World Health Organization categorizes diseases in three types, according to the following criteria: (I) diseases which affect both populations of high-income countries and populations of countries with lower incomes (for instance, diabetes, cardiovascular diseases, asthma, and chronic obstructive pulmonary disease); (II) diseases which affect rich and poor countries, but the cases are concentrated more heavily in poor countries. There is allocation of investments for these conditions, but this is not consonant with the disease's burden (examples are HIV, dengue, and diarrheal diseases); (III) diseases concentrated almost entirely in developing countries, and which receive few resources for research (for example, Chagas disease, malaria, and leprosy; WHO, 2012a; Moran, 2013).

Our results showed that the diseases studied the most in Latin America and the Caribbean are in category I. Regarding diseases found in categories II and III, there were few studies. This shows that although there has been investment into researching diseases such as Chagas disease and gastrointestinal tract infections, it has as yet been insufficient.

The non-communicable diseases which are chronic diseases, such as cardiovascular diseases, diabetes, cancer, and chronic respiratory diseases are related to the deaths of 40 million people per year worldwide, corresponding to 70% of deaths. The 2030 Agenda of the WHO for Sustainable Development considers the control of these conditions to be a major challenge, and prioritizes the investments in the prevention and reduction of deaths (WHO, 2017b). In countries such as Argentina, Chile, Paraguay, and Uruguay, 77% of deaths of men, and 83% of deaths of women, in 2013, were due to the non-communicable diseases (Legetic et al., 2016).

A number of factors have contributed to the increase in the mortality rates from non-communicable diseases in Latin America and the Caribbean. These include the aging of the population, the urbanization process, and greater exposure to risk factors such as smoking, obesity, stress, and the consumption of industrialized foods. In these regions, the cardiovascular diseases are responsible for 31% of deaths (Barreto et al., 2012; Stringhini and Bovet, 2017). The social determinants of health, such as socioeconomic or educational level, or access to the health services, also influence individuals' vulnerability to these chronic conditions (Legetic et al., 2016). The promotion of public policies aimed at reducing these risk factors, and at improving access to healthcare, could be an important step for reducing the impact of these non-communicable diseases (Anauati et al., 2015).

The 10/90 gap in medical and health-related research may not be as current, because the transition from transmissible diseases to the non-communicable conditions among the leading causes of death in less developed countries can have reduced the unequal in investment in health research.

According to the present study's results, international clinical trials are in alignment with the health needs of Latin American and Caribbean countries, as one can observe that among the diseases studied most are diabetes, asthma, and cancer. According to the World Health Organization, chronic obstructive pulmonary disease, which was one of the conditions investigated the most, is expected to be the third greatest cause of death worldwide in 2030 (WHO, 2017c). This disease has been reported as one of the main causes of death in countries such as Argentina, Uruguay, and Colombia (Rubinstein et al., 2011; WHO, 2017d).

The transmissible diseases, such as tuberculosis, AIDS, and dengue, which have an important impact on public health in the region of Latin America and the Caribbean, have as yet aroused little interest among institutions which finance research. Between 1995 and 2010, there was an increase in the incidence of dengue in this region. In 2016, 1,032 deaths occurred in the Americas due to infection by dengue. In that same year, Brazil recorded 1.5 million cases of dengue—three times the number of cases recorded in 2014 (Ramos-Castañeda et al., 2017; WHO, 2017e). In Latin America and the Caribbean, ~30% of disability-adjusted life years are attributed to transmissible diseases (Barreto et al., 2012).

Each year, the neglected diseases cause ~534,000 deaths, and a burden of disease of 57 million disability-adjusted life years worldwide. Approximately 9% of this burden of disease is related to the region of Latin America and the Caribbean. One of the objectives of the Millennium Development Goals, established by the United Nations, is the elimination of certain neglected diseases, such as Chagas disease, leprosy, leishmaniasis, and filariasis (Kappagoda and Ioannidis, 2014; WHO, 2015a, 2017d; PAHO, 2017a).

According to results, the industry has sponsored clinical trials for chronic conditions which affect the populations of both poor and rich countries. Universities, on the other hand, have sponsored clinical trials investigating new drugs for treating conditions—such as gastrointestinal infections—which disproportionately affect countries with fewer resources. It must be noted, however, that universities sponsor few clinical trials when compared with the industry. Gastrointestinal infections are generally associated with shortcomings in basic sanitation and infrastructure. The diarrheal diseases continue to be a public health problem in the region of Latin America and the Caribbean; there continues to be a high mortality rate for children below 5 years old due to these conditions. In 2015, in Haiti, Nicaragua, Guatemala and Bolivia, mortality among children below 5 years old due to diarrhea corresponded to 10, 8, 7, and 6%, respectively (Overgaard et al., 2012; Fletcher et al., 2013; Pinzón-Rondón et al., 2015; PAHO, 2017b).

University research is extremely important for the development of new drugs. Innovations from universities tend to be in line with the aspiration to improve global access to medicines. In the United States, institutions of higher education have been encouraged to adopt official resolutions to the effect that the global improvement of human welfare is the highest goal of university technology transfer. To comply with this requirement, universities are encouraged to sign up to licensing arrangements that promote access to their health-related innovations in less-developed countries, to boost research into the neglected tropical diseases, and to collaborate with public and private research bodies in low- and middle-income countries seeking to produce drugs specifically targeting these diseases (Chokshi, 2006).

The US government leads both research and funding for trials involving 26 of the 30 most neglected diseases (the four areas in which it has a lesser role are meningitis, bacterial pneumonia, dengue fever, and Buruli ulcer) (Roehr, 2012). The present study showed that governmental institutions have financed studies on conditions that affect the countries of Latin America and the Caribbean, such as HIV and malaria (Barreto et al., 2012). However, the results also showed that the clinical trials involving, for example, Chagas disease, were financed by nongovernmental and nonprofit organizations and by a hospital. Although Chagas disease has been studied, the number of studies undertaken, and the amount of resources allocated for research, remains negligible. The increase in investment in research into neglected diseases in recent years is due mainly to contributions from foundations (WHO, 2012b).

In the region of Latin America and the Caribbean, ~7,000 deaths occur per year due to Chagas disease. This condition has been related to significant health costs. In 2013, the cost related to this condition was estimated at $7 billion. In Brazil, the cost referent to hospitalizing patients with chagasic cardiomyopathy has been estimated at $467 per day, higher than the cost of inpatient treatment due to heart failure (WHO, 2015b). The number of cases of this disease is highest in the following countries: Argentina (376,309), Brazil (231,364), Colombia (131,388), and Bolivia (121,437) (WHO, 2015c).

Pharmaceutical companies have contributed to treating populations affected by the neglected diseases through donating essential drugs. This has improved the patients' living conditions. Nevertheless, these companies continue to direct few resources toward research and development into new treatments for these neglected diseases (WHO, 2017f).

There has been reluctance on the part of drug companies to develop drugs for neglected diseases, because the patients afflicted by these also tend to lack money to pay for drug treatment, which removes the financial incentive. Accordingly, two problems needing to be resolved are: difficulty accessing drugs which have been developed, and the funding of research into neglected diseases (Chokshi, 2006).

In spite of this advance in the increase of investments in research into neglected diseases, only 4.3% of expenditure on research worldwide has been directed toward the health needs of developing countries. According to specialists in financing research, local governments should ringfence 2% of their budget for research activities in essential health—while countries with more resources should help to finance research into areas of interest to poor countries, and provide support in order to improve their research capability (WHO, 2012b).

The neglected diseases are related to conditions of poverty and social and health inequalities. As a result, local governments have a fundamental role in the control and eradication of these diseases, based on the prioritization of policies for reducing poverty, improving infrastructure, and mobilizing communities in programs designed to control disease vectors (Manderson et al., 2009).

First in human trials have not been common in low- and middle-income countries due to the poor infrastructure for research and clinical practice, as well as below-standard institutional capacity and toothless regulatory agencies (Kapiriri et al., 2011). There are also regulatory requirements that may impact on the conduct of phase 1 trials; for example, in Argentina, only institutions qualified to provide inpatient treatment may conduct phase I studies under regulations implemented by the Health Ministry (ANMAT, 2010). In Brazil, the latest clinical trials regulation published by the Brazilian Health Regulatory Agency (Resolution N. 9/2015), established that research projects must be evaluated within 90 days, but that phase 1 and 2 clinical trials, which are considered more complex, should be evaluated within 180 days (Brazilian Health Regulatory, 2015).

The presence of data monitoring committees is strongly recommended in clinical studies that have substantial safety issues, in order to provide added protection for the vulnerable populations that are often enrolled in studies, which include children and pregnant women, as well as the elderly and other populations considered to be particularly vulnerable (FDA, 2006). In Brazil, the monitoring of phase III studies by these committees is required based on the publication of Resolution N. 9 of February 20th 2015 (Brazilian Health Regulatory, 2015). The majority of trials conducted in these countries are monitored by Data Monitoring Committees, but this does not mean that studies have been conducted according to good clinical practices, or that all studies involving vulnerable populations have been monitored. In Brazil, for example, one study that involved participants (Age ≥ 1 year and < 18 years) with purpura and other hemorrhagic conditions was found not to have been monitored by a data monitoring committee.

Few studies involving the pediatric population have been undertaken in the countries addressed in this paper. Considering that there are conditions that are rare in this population, the challenge is to recruit patients in studies. By far most drugs on the market are not authorized for use in children; neither has adequate research, evidencing these drugs' safety and efficacy in this population, been conducted (WHO, 2007; Gamboa and Gregianin, 2013; Helmchen et al., 2014).

## Limitations

Our search may not have captured all trials conducted in Latin America in 2014. The ICTRP accepts trial records from data providers only if evidence is given that said records were created and managed in line with WHO registry criteria. In the Latin America and the Caribbean region, only the Brazilian Clinical Trials Registry (ReBec) and the Peruvian Clinical Trials Registry (REPEC) are data providers. Therefore, the WHO trial registry cannot contain all trials conducted in Latin America and the Caribbean region, which may compromise the study of the current scenario of clinical trials in this region (WHO, 2017g).

Also, at the time of writing, the ICTRP accepts data from 16 registries, both national and regional, that are known to comply with specified quality criteria (WHO, 2017g). However, clinical trials may be registered in other registries, such as those under the auspices of the pharmaceutical industry.

## Conclusions

The international multicenter clinical trials investigating new drugs are aligned with the health needs of the region of Latin America and the Caribbean, when one considers the burden of the Non-communicable diseases in this region. However, the transmissible diseases, such as tuberculosis and AIDS, and the neglected diseases, such as Chagas disease and dengue, which have an important impact on public health in this region, continue to arouse little interest among the institutions which finance the clinical trials.

It may be that the fact that there are few phase I and II studies in Latin America is related to a lack of adequate infrastructure for supporting clinical sites to conduct studies in accordance with Good Clinical Practice and regulatory requirements.

Most clinical trials in Latin America and the Caribbean are monitored by data monitoring committees. The sponsors of the studies, regulatory agencies and ethics committees should work such that these data monitoring committees may be engaged and monitor the studies independently.

Clinical trials involving the pediatric population are still a minority. Therefore, although it is necessary for countries to create incentives for conducting studies in this population, it is also necessary to guarantee the participants' rights and well-being, in particular because this population is considered vulnerable.

## Author contributions

RS, MC and MN made substantial contributions to conception, design and acquisition of data. In addition to they analyzed and interpreted the data. AA and DG made substantial contributions to conception and design. All authors read and approved the final manuscript.

### Conflict of interest statement

The authors declare that the research was conducted in the absence of any commercial or financial relationships that could be construed as a potential conflict of interest.
